# Gray and white matter alterations in different predominant side and type of motor symptom in Parkinson's disease

**DOI:** 10.1111/cns.13877

**Published:** 2022-06-07

**Authors:** Jingwen Chen, Xianchen Jiang, Jingjing Wu, Haoting Wu, Cheng Zhou, Tao Guo, Xueqin Bai, Xiaocao Liu, Jiaqi Wen, Zhengye Cao, Luyan Gu, Wenyi Yang, Jiali Pu, Xiaojun Guan, Xiaojun Xu, Baorong Zhang, Minming Zhang

**Affiliations:** ^1^ Department of Radiology, The Second Affiliated Hospital Zhejiang University of Medicine Hangzhou China; ^2^ Quzhou Center for Disease Control and Prevention Quzhou China; ^3^ Department of Neurology, The Second Affiliated Hospital Zhejiang University of Medicine Hangzhou China

**Keywords:** laterality, magnetic resonance imaging, motor types, Parkinson's disease, tract‐based spatial statistics

## Abstract

**Background:**

Parkinson's disease (PD) is highly heterogeneous reflected by different affected side of body and type of motor symptom. We aim to explore clinical characteristics and underlying brain structure alterations in PD with different predominant sides and motor types.

**Methods:**

We recruited 161 PD patients and 50 healthy controls (HC). Patients were classified into four subtypes according to their predominant side and motor type: left akinetic/rigid‐dominant (LAR), left tremor‐dominant (LTD), right akinetic/rigid‐dominant (RAR), and right tremor‐dominant (RTD). All participants assessed motor and cognitive performances, then underwent T1‐weighted and diffusion tensor imaging scanning. A general linear model was used to compare neuroimaging parameters among five groups.

**Results:**

Among four PD subtypes, patients of LAR subtype experienced the worst motor impairment, and only this subtype showed worse cognitive performance compared with HC. Compared with HC and other subtypes, LAR subtype showed a significant reduction in cortical thickness of the right caudal‐anterior‐cingulate gyrus and fractional anisotropy of the right cingulum bundle.

**Conclusions:**

We demonstrated that LAR subtype had the worst clinical performance, which the severer damage in the right cingulate region might be the underlying mechanism. This study underscores the importance of classifying PD subtypes based on both the side and type of motor symptom for clinical intervention and research to optimize behavioral outcomes in the future.

## Graphical abstract

The predominant side and type of motor symptoms contribute to clinical difference in Parkinson's disease. Patients with predominant left‐side akinesia/rigidity subtype performed worst in motor and non‐motor symptoms. The underlying mechanism might be the most severe damage in cingulate region in the right hemisphere.

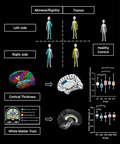

## 1 INTRODUCTION

Parkinson's disease (PD) is the second most prevalent neurodegenerative disorder, which is characterized by progressive loss of dopaminergic neurons in the nigrostriatal system and results in motor disturbances, including bradykinesia, rigidity, and resting tremor.^1^, ^2^, ^3^ Researches have demonstrated that different types of motor symptoms (generally, akinesia/rigidity‐dominant [AR], and tremor‐dominant [TD] type) lead to diverse clinical prognoses.^4^, ^5^, ^6^, ^7^, ^8^, ^9^ Additionally, PD is often presented as asymmetric motor involvement in the initial course and may last throughout the whole period.^10^, ^11^ PD patients with predominant left‐side or right‐side motor symptoms reflect a degenerative process asymmetrically affecting the two hemispheres may indicate different prognoses either.^12^, ^13^, ^14^, ^15^ Preliminary studies suggest that there is an intricate relationship between laterality and type of motor symptoms and the combination of these factors may influence motor progression and cognitive deterioration.^16^, ^17^, ^18^, ^19^ Further exploration is worthwhile to discover the complex brain alterations that underlie these differences, which can deepen our perception of the underlying mechanisms corresponding to clinical prognosis.

Numerous studies suggested that patients presenting with TD have a slower motor progression than those with AR type.^4^, ^7^, ^20^ As for the non‐motor symptoms, the AR type is concerned with a faster rate of cognitive decline and a higher incidence of dementia.^7^, ^21^ In addition, the influence of predominant side of motor symptoms also has been reported. Results suggested that PD patients served milder motor impairment on their dominant side.^10^, ^22^ Other research reported that patients who favored the right hand but had served motor impairment at the left side were associated with faster symptom progression and worse outcomes in multiple clinical domains.^23^, ^24^, ^25^ Interestingly, when concerned about the laterality and type of symptoms concurrently, most studies agreed that PD patients with the right‐side tremor showed intact cognition compared with the right‐side AR and left‐side TD/AR subtypes,^16^, ^19^, ^24^ while the left‐side AR subtype may have a worse prognosis.^15^ Thus, a complex relationship exists between these two factors and may influence the disease progression in turn. Nevertheless, the mechanism behind this remains unclear.

Structural magnetic resonance imaging (MRI) such as T1‐weighted imaging and diffusion tensor imaging (DTI) have been used to investigate neurodegeneration disease extensively, which provide us with changes in gray and white matters that can help reveal the mechanisms behind different clinical manifestations and outcomes.^26^ Various structural MRI modals have applied for exploring potential differences in PD subtypes^27^; for instance, researches based on T1 imaging showed smaller gray matter particularly in areas involving motor, cognitive, limbic, and associative functions in patients with AR type.^28^ Similarly, a few DTI studies have revealed that AR type patients had greater diffusivities and greater reduction in white matter integrity in basal ganglia and out of nigrostriatal tracts.^29^, ^30^ On the other hand, studies with regard to laterality of PD suggest that patients with predominant the left‐side or right‐side motor symptoms may be associated with lateralized gray matter atrophy and white matter damage in the contralateral hemisphere.^31^

Nonetheless, scarcely any MRI studies have examined the influence of side and type of motor symptom on the presence of motor and non‐motor symptoms in PD so far. Investigations using combined brain imaging modalities may shed light on the alterations of brain structure in PD with different predominant sides and motor types and provide potential avenues for prognosis and intervention of the corresponding PD.

## 2 SUBJECTS AND METHODS

This study was in accordance with the approval of the Medical Ethic Committee of Second Affiliated Hospital of Zhejiang University School of Medicine and all patients were informed consent forms.

### 2.1 Subjects

We recruited 262 right‐handed PD patients and 50 right‐handed healthy controls (HCs). Subjects in the PD and HC groups were matched for age and gender. The patients were diagnosed by an experienced neurologist according to UK Parkinson's Disease Society Brain Bank criteria.^32^ The Unified Parkinson's Disease Rating Scale (UPDRS) and Hoehn and Yahr stages were used to evaluate the disease severity of PD patients. For patients on medication, clinical evaluations, and image scanning were carried out after withholding anti‐parkinsonian medicine overnight (at least 12 h). Scales of the Mini‐Mental State Examination (MMSE), the Hamilton Depression Scale (HAMD), and the Hamilton Anxiety Scale (HAMA) were also obtained from all subjects.

A total of 14 participants were excluded with a history of cerebral trauma (*n* = 2), psychiatric or neurological disorders (except PD for the patients) (*n* = 1), abuse of alcohol, insulin‐dependent diabetes (*n* = 3). And patients had poor imaging quality (*n* = 4) and incomplete clinical data (*n* = 4) were also excluded.

**FIGURE 1:**
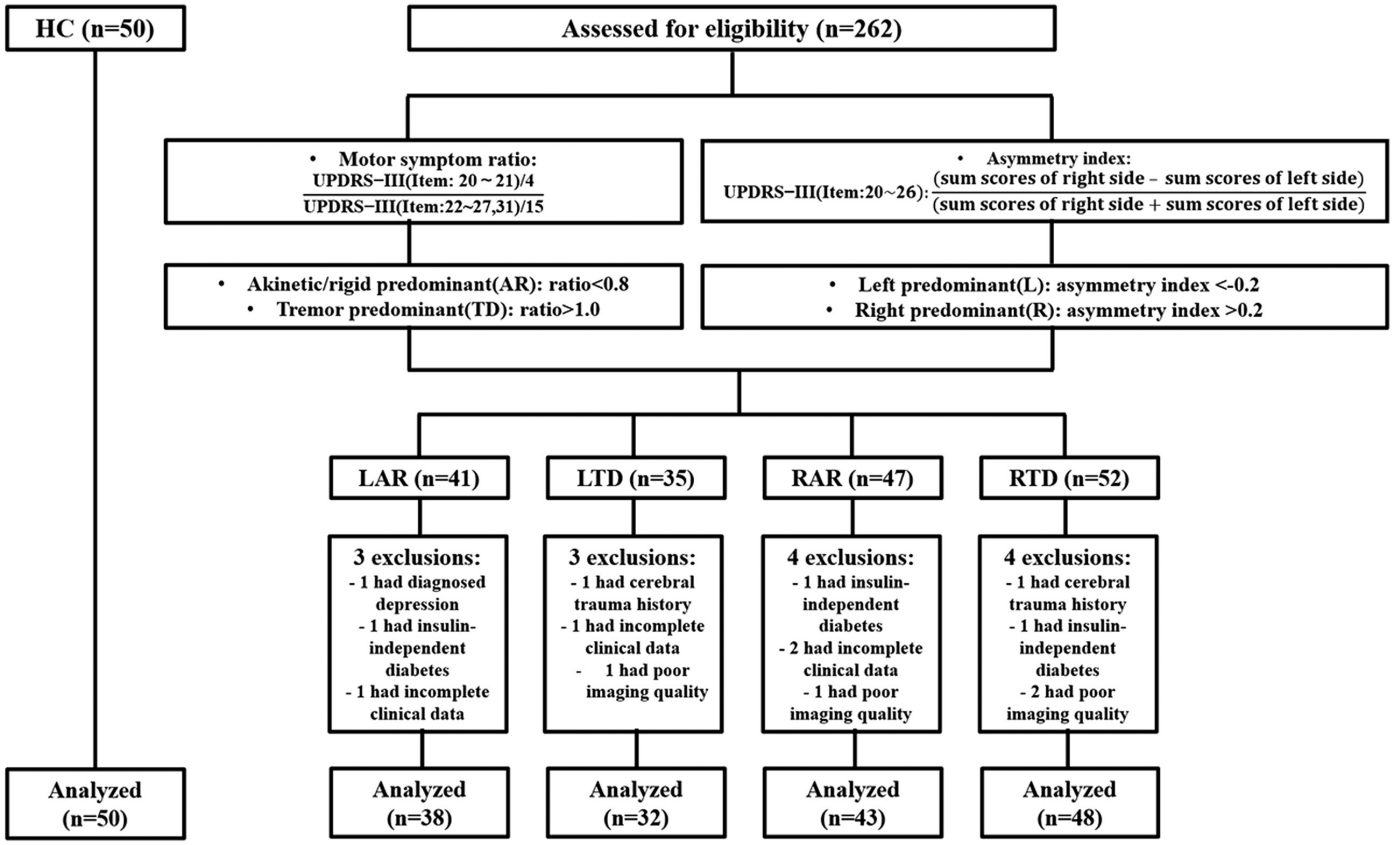
Patients flow diagram of inclusion and exclusion

Patients were classified into four subtypes according to their predominant side and type of motor symptoms: left akinetic/rigid‐dominant (LAR), left tremor‐dominant (LTD), right akinetic/rigid‐dominant (RAR), and right tremor‐dominant (RTD). The predominant side of each patient was identified by following steps according to previous studies.^25^, ^33^ First, the sum of the scores of UPDRS‐III items 20 to 26 was obtained primarily for each side of the body. Then, the UPDRS asymmetry index was calculated with the following formula:

$$\frac{\left(\mathrm{sum}\;\text{scores of right side}-\mathrm{sum}\;\text{scores of left side}\right)}{\left(\mathrm{sum}\;\text{scores of right side}+\mathrm{sum}\;\text{scores of left side}\right)}$$

and patients were defined as the right‐side predominant group if the index was >0.2 and as the left‐side predominant group if the index was <−0.2.^34^, ^35^ As for the predominant type of motor symptoms, the mean UPDRS‐III tremor scores (calculated as the sum of items 20 and 21 divided by 4) and the mean UPDRS‐III akinetic/rigid scores (calculated as the sum of items 22–27 and 31 divided by 15) were gained for each patient.^36^, ^37^ Furthermore, patients were grouped into the TD type if the ratio was >1.0 and into the AR type if the ratio was <0.8 based on the ratio of tremor score to akinetic/rigid score. The patients who did not meet the criteria were excluded for they had no laterality and symptom preference (*n* = 87). Finally, a total of 161 PD patients (including 38 LAR, 32 LTD, 43 RAR, and 48 RTD) and 50 HC were enrolled in this study (Figure 1).

### 2.2 MRI data acquisition and processing

All subjects were scanned in a 3.0 Tesla MRI machine (GE Discovery 750) equipped with an 8‐channel head coil. During MRI scanning, the heads of subjects were stabilized with restraining foam pads, and the subjects were told to stay still with their eyes closed.

#### 2.2.1 Structural imaging

Three dimensions T1‐weighted images were acquired using a Fast‐Spoiled Gradient Recalled sequence: repetition time (TR) = 7.336 ms; echo time (TE) = 3.036 ms; inversion time = 450 ms; flip angle = 11°; field of view (FOV) = 260 × 260 mm^2^; matrix = 256 × 256; slice thickness = 1.2 mm; 196 continuous sagittal slices.

The T1‐weighted images were pre‐processed using the default command “recon‐all” provided in the FreeSurfer (v6) pipeline (http://surfer.nmr.mgh.harvard.edu/). The “recon‐all” procedure was used to perform its full reconstruction pipeline, which provided subcortical segmentation and volume measurement, in addition to cortical reconstruction, and measurement of cortical thickness. The pre‐processing pipeline involved non‐uniform intensity normalization, Talairach transform computation, intensity normalization, skull stripping, subcortical segmentation, intensity normalization (input was the brain volume without the skull), white matter segmentation, tessellation (of the gray and white matter boundary), automatic topology correction, creation of final surfaces (pial and white matter), and parcellation (creation and measurement).^38^, ^39^

Finally, the cortical thickness and subcortical volume features were extracted according to the boundaries and labeling of the Desikan–Killiany atlas, which contains 34 cortical regions of interest (ROIs) in each of the individual hemispheres.

#### 2.2.2 Diffusion tensor imaging

Diffusion tensor imaging images were acquired using a spin echo‐echo planar imaging sequence with 30 gradient directions (b value = 1000 s/m^2^): repetition time = 8000 ms; echo time = 80 ms; flip angle = 90°; field of view = 256 × 256; matrix = 128 × 128; slice thickness = 2 mm; slice gap = 0 mm; 67 interleaved axial slices.

Diffusion‐weighted images were processed using FMRIB Diffusion toolbox (FSL, http://www.fmrib.ox.ac.uk/fsl/). The skulls were stripped from the T1‐weighted image and DTI images for each participant first. Then, eddy currents and head‐motion artifacts in diffusion data were corrected. Finally, diffusion parameters (fractional anisotropy [FA], and mean diffusivity [MD]) were calculated.

In order to perform Tract‐based spatial statistics (TBSS),^40^ FA maps of all the subjects were further processed. Firstly, erosion was performed with tbss_1_preproc. Secondly, the eroded FA maps were nonlinearly registered to FA template in Montreal Neurological Institute (MNI) space with tbss_2_reg. Then, the mean of all FA images was calculated and skeletonized with tbss_3_postreg. To exclude regions characterized by high cross‐subject variability and bad alignment, the mean skeleton was thresholded to 0.2, and skeletonized FA maps were derived for each subject, by projecting the FA images onto the skeleton with tbss_4_prestats. MD maps were registered to MNI standard space, by using the same parameters used to register the FA image to the FA template in MNI space. MD data were obtained with tbss_non_FA.

Finally, to obtain the white matter tract segmentation, the Johns Hopkins University tractography atlas was used, which contains 20 main white matter tracts.^41^ Mean FA and MD values were computed in each white matter ROI in white matter skeleton (standard space) for each participant.

### 2.3 Statistics analysis

Demographic and clinical variables were analyzed by applying Statistical Product and Service Solutions (version 26.0). The Kolmogorov–Smirnov test was used to determine whether the quantitative variables were normally distributed. Accordingly, quantitative variables with normal distribution were presented as means ± standard deviations (SD) and compared by analysis of variance (ANOVA). And quantitative variables with nonnormal distribution were presented as medians with ranges and compared by the Kruskal–Wallis test. All qualitative variables were analyzed by using the χ^2^‐test. Post hoc analysis was also performed for multiple comparisons.

Statistical analyses for cortical thickness, subcortical volume (divided by total intracranial volume), and diffusion variables (FA and MD) were conducted using MATLAB (R2019a). A general linear model (GLM) with age, gender, and education as covariates was used to compare structural neuroimaging differences among the five groups. In ROI analyses, the false discovery rate (FDR) correction with a threshold at *p* < 0.05 was used for comparisons across multiple ROIs.

## 3 RESULTS

Demographic and clinical data for each of the four PD subtypes and HC were presented in Table 1. No significant differences were found in age (*p* = 0.834) and gender (*p* = 0.149) among five groups. And four PD subtypes did not show significantly different disease duration (*p* = 0.458) and total levodopa equivalent daily dose (LEDD) (*p* = 0.904). Significant differences were found in UPDRS‐total scores (*p* = 0.004), UPDRS‐III scores (*p* = 0.003) and Hoehn and Yahr stages (*p* = 0.002) among four PD subtypes, the post hoc analyses showed the scores were higher in the LAR subtype than other PD subtypes. Significant differences were also found in HAMD (*p* < 0.001) and HAMA (*p* = 0.008) scales. The post hoc analyses showed higher scores in four PD subtypes compared with HC, but there were no differences among four PD subtypes. The education was significantly different in five groups (*p* = 0.043), which post hoc analyses showed the education years were slightly lower in LAR and LTD than RAR subtype. The difference in MMSE was not statistically significant (*p* = 0.079), although post hoc analyses revealed the LAR subtype performed worse than HC and RAR subtype. Since education was strongly correlated with performance on cognitive measures and was therefore selected as a covariate together with age and gender for the neuroimaging analyses.

**TABLE 1:** Demographic and clinical data for each of the four PD subtypes and HC

	HC (*n* = 50)	Left side		Right side		*p*‐Value	Post hoc
		PD‐AR (*n* = 38)	PD‐T (*n* = 32)	PD‐AR (*n* = 43)	PD‐T (*n* = 48)		
Age	59.2 ± 6.3	59.4 ± 10.3	60.4 ± 8.9	57.9 ± 10.6	59.3 ± 9.7	0.834	
Gender (M/F)	28/22	17/21	16/16	25/18	34/14	0.149	
Education	8.5 ± 3.2	6.9 ± 4.9	7.7 ± 4.3	9.8 ± 4.7	8.3 ± 4.4	**0.043**	f, h
Disease duration	/	4.5 ± 4.5	3.9 ± 3.1	3.4 ± 3.2	4.5 ± 4.2	0.458	
LEDD	/	293.4 ± 261.8	310.5 ± 252.1	284.1 ± 211.4	268.9 ± 268.6	0.904	
UPDRS‐total	/	38.1 ± 18.6	27.1 ± 13.0	28.1 ± 12.0	27.7 ± 16.0	**0.004**	e, f, g
UPDRS‐I	/	1.6 ± 1.8	1.4 ± 1.5	1.1 ± 1.2	1.4 ± 1.5	0.450	
UPDRS‐II	/	9.8 ± 5.9	7.4 ± 4.8	8.2 ± 3.8	7.9 ± 5.0	0.205	
UPDRS‐III	/	25.4 ± 13.3	17.7 ± 8.6	17.9 ± 8.7	17.6 ± 11.4	**0.003**	e, f, g
UPDRS‐IV	/	1.3 ± 2.0	0.6 ± 0.9	0.7 ± 1.3	0.8 ± 1.3	0.198	e
H&Y staging	/	2.4 ± 0.4	2.0 ± 0.5	2.1 ± 0.6	2.0 ± 0.6	**0.002**	e, f, g
MMSE	28.0 ± 1.7	26.2 ± 4.0	26.9 ± 3.7	27.7 ± 2.5	27.2 ± 3.3	0.079	a, f
HAMD	2.5 ± 3.7	7.5 ± 6.9	6.3 ± 4.4	5.9 ± 5.5	6.9 ± 5.9	**<0.001**	a, b, c, d
HAMA	3.2 ± 4.2	6.3 ± 5.9	7.0 ± 5.2	5.8 ± 5.4	6.2 ± 5.4	**0.008**	a, b, c, d

*Note:* Values are mean ± SD. Bold, *p* < 0.05, significantly different between groups. Post hoc analyses: a: HC vs. PD‐LAR; b: HC vs. PD‐LTD; c: HC vs. PD‐RAR; d: HC vs. PD‐RTD; e: PD‐LAR vs. PD‐LTD; f: PD‐LAR vs. PD‐RAR; g: PD‐LAR vs. PD‐RTD; h: PD‐LTD vs. PD‐RAR; i: PD‐LTD vs. PD‐RTD; j: PD‐RAR vs. PD‐RTD.

Abbreviations: HAMA, Hamilton Anxiety Scale; HAMD, Hamilton Depression Scale; HC, healthy controls; LAR, left akinetic/rigid‐dominant; LEDD, levodopa equivalent daily dose; LTD, left tremor‐dominant; MMSE, Mini‐Mental State Examination; PD, Parkinson's disease; RAR, right akinetic/rigid‐dominant; RTD, right tremor‐dominant; UPDRS, Unified Parkinson's Disease Rating Scale.

### 3.1 Comparison of T1‐weighted and diffusion tensor images

**FIGURE 2:**
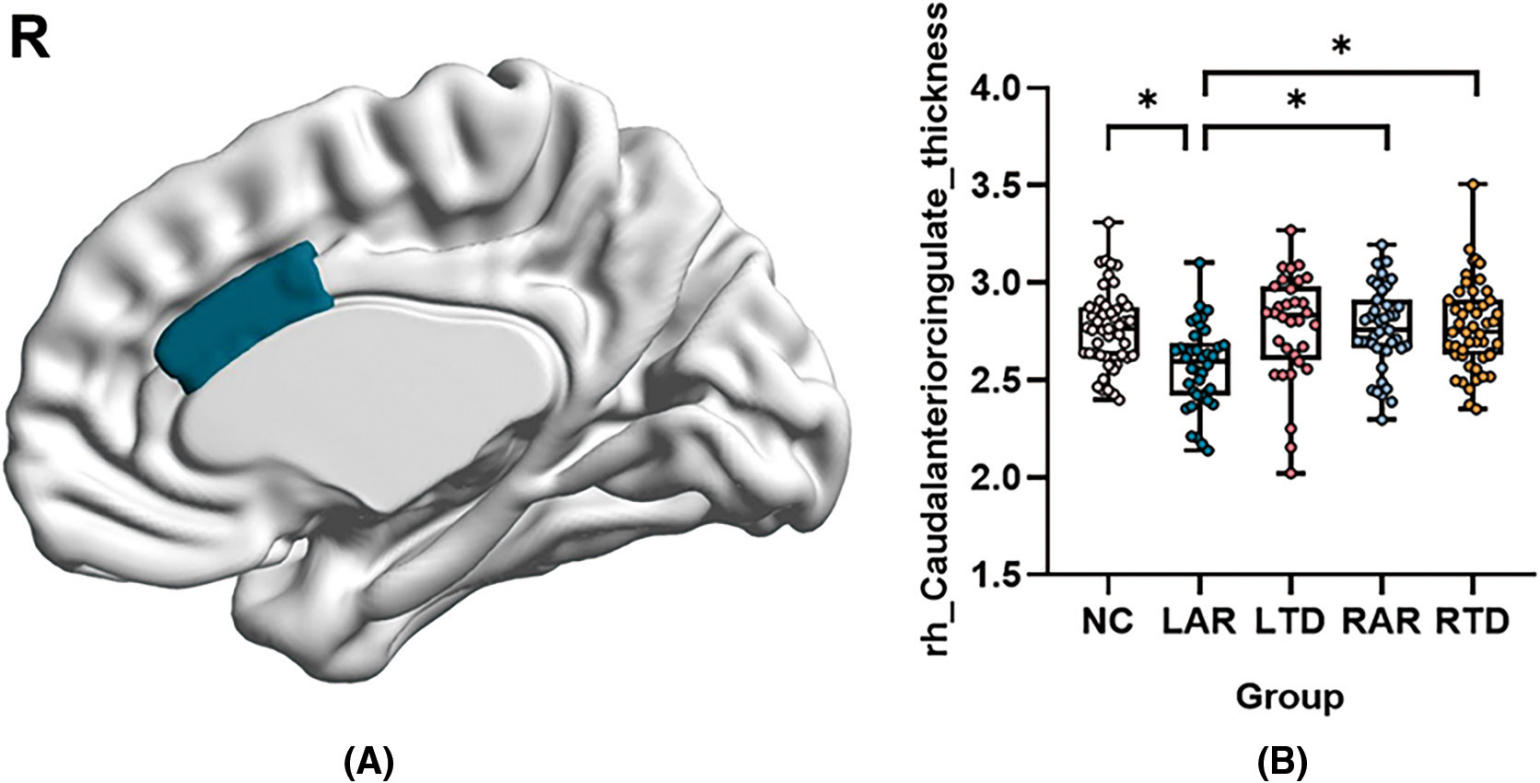
Comparison of cortical thickness among five groups. Comparison of cortical thickness among five groups showed significant difference in caudal‐anterior cingulate in the right hemisphere (A). False discovery rate (FDR) correction was used for multiple comparisons and statistical significances were represented as: *p < 0.05 (B)

A significant difference of cortical thickness was found in caudal‐anterior‐cingulate gyrus in the right hemisphere (*p* = 0.009) in five groups, and further post hoc analyses with FDR correction showed that LAR subtype had significant atrophy worse than the HC (*p* = 0.013), RAR subtype (*p* = 0.045) and RTD subtype (*p* = 0.035) (Figure 2). No significant difference was detected between LAR subtype and LTD subtype.

**FIGURE 3:**
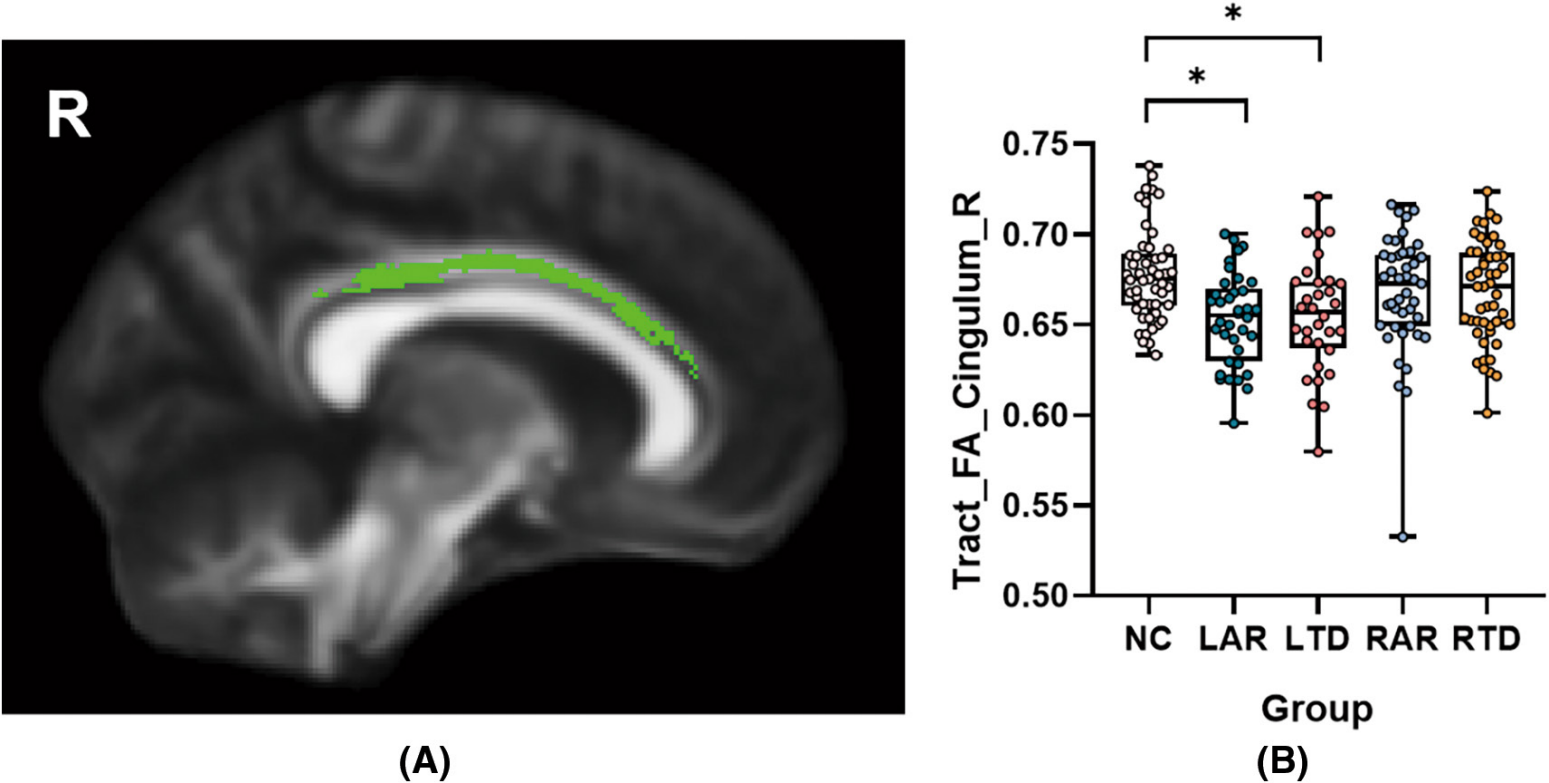
Comparison of mean fractional anisotropy (FA) values among five groups. Comparison of mean FA values of WM tracts among five groups showed significant difference in cingulum bundle in the right hemisphere (A). False discovery rate (FDR) correction was used for multiple comparisons and statistical significances were represented as: *p < 0.05 (B)

The mean FA values of cingulum bundle in right hemisphere remained significantly different among five groups after FDR correction (*p* = 0.009) (Figure 3). Post hoc analyses showed LAR subtype obtained the lowest mean FA values among five groups, which was significantly lower than HC (*p* = 0.010). Likewise, the LTD subtype had lower mean FA values than HC (*p* = 0.011). No significant differences in mean FA values were detected between RAR, RTD subtypes, and HC. While five groups did not show significant differences in mean MD values in any regions.

## 4 DISCUSSION

In this study, we found that PD patients characterized by different combinations of predominant‐side and predominant‐motor type (i.e., LAR, LTD, RAR, and RTD) served diverse severity of both motor and non‐motor symptoms. Among all four PD subtypes, patients in LAR subtype had the worst motor manifestations, and only this subtype showed worsened cognitive performance compared with HC. Further brain structure comparison detected this subtype had significantly worse damage in gray and white matter of the right cingulate region, providing structure evidence of their worst symptoms.

Consistent with Riederer and his colleagues' findings summarizing that the LAR subtype had a poorer prognosis,^15^ our results demonstrated that patients in this subtype had worst motor impairments relative to other patients, and only them showed decreased cognitive performance relative to HC. The influence of laterality of motor symptoms has been confirmed in previous studies. Primarily, the healthy brain appears to have an asymmetric dopamine distribution, with higher dopamine contents in the left than the right striatum.^42^ This neurochemical asymmetry renders the right striatum relatively more vulnerable to the effects of dopaminergic denervation in PD and leads to disorders in the left‐side body.^43^ Furthermore, they suggested that dominant side had a protective effect due to enhanced exercise which in turn caused better synaptic plasticity in the dominant hemisphere.^31^, ^44^ As all patients enrolled in our study were right‐handed subjects, which reflected their left hemisphere dominance, they might had better synaptic plasticity in the left hemisphere to overcome the pathological alternation in PD with the right‐side motor symptoms. Moreover, more distribution of striatal dopamine in left hemisphere enhanced connections to the cortex to make it more robust in face of damage and therefore followed positive consequences in terms of symptoms.^24^ Therefore, we speculated that right‐handed patients predominating with the right‐side motor symptoms (damage in the left hemisphere) performed milder severity compared with those predominating with left‐side (damage in the right hemisphere), just as shown in previous research.^44^ In the future, it might be possible to combat disease progression by strengthening exercises in the more affected side of the body. Effect of motor types should also be noted. Previous studies had confirmed that AR type was related to a more dopaminergic deficiency in nigrostriatal projection pathway compared with TD type.^45^, ^46^ Except for it, PD patients with predominant AR symptom have been reported undergoing degenerating across more brain regions.^5^, ^8^, ^47^ Both two factors played an important role in worsening performance of PD with predominant AR symptom. While a few studies did not find significant differences in motor manifestations among PD characterized by different side and type of motor symptoms.^16^, ^17^, ^18^, ^19^, ^48^ These might be caused by two factors. First, most of these studies aimed at non‐motor features of four PD subtypes, so motor severity across subtypes was controlled at the same level in order to remove potential effect of motor impairment. Second, different criteria of classification for lateralization could also influence results. Previous studies used the side of disease onset or initial motor symptom as the criterion for classification. While as the course of disease progresses, the original performance could no longer be suitable for representing the present manifestation. And the classification of predominant‐side and predominant‐motor type within current performance can better reflect the current clinical manifestations. Our results revealed the combination of predominant‐side and predominant‐motor symptom could affect performance of PD patients. Given the severer symptoms in patients of LAR subtype, more attention should be paid to this subtype in future research and more support should be given to these individuals in clinical practice.

Meanwhile, this study identified the brain structural changes underlying clinical differences in PD with different predominant‐side and motor type. The structural MRI revealed atrophy in the right caudal‐anterior cingulate cortex (ACC) and damaged integrity in the right cingulum bundle in left‐side predominant PD patients, particularly in LAR subtype. ACC is an integrated hub for information processing and regulation, and human neuroimaging studies have recognized its existence of motor, cognitive and affective subdivisions.^49^, ^50^ Thereinto, caudal division of ACC is more relevant to sensorimotor regions, whereas the rostral division is associated more with prefrontal regions.^51^ Ulteriorly, the cingulum bundle, known as projection as well as association fibers, traverses the frontal, parietal, and temporal cortices, allowing cingulate, noncingulate frontal, and subcortical fibers to reach both cingulate and other limbic targets.^52^, ^53^ In light of previous studies,^53^ we deduced that the worst motor impairment in the LAR subtype may result from the damaged right cingulate bundle connecting the medial side of the supplementary motor area (SMA) complex to the injured cingulate motor areas in the ipsilateral hemisphere. As is well known that cingulate gyrus is considered to be more related to cognitive functions just as we observed in numerous researches focusing on non‐motor symptoms in PD.^54^, ^55^ Studies using DTI have shown that FA of the cingulate bundle was significantly lower in patients with cognitive decline for reduced FA indicated damage to the fiber bundles. Furthermore, reduced FA of anterior cingulate bundle was associated with MMSE.^56^, ^57^ In addition, damage in cingulum bundle was a biomarker of mild cognitive impairment in Alzheimer's disease,^58^ given the pathologic association of total tau, the LAR subtype might be at risk for developing cognitive impairment. Therefore, early awareness of LAR subtype is critical for prognosis and allows those patients for closer monitoring and management.

### 4.1 Limitations

The limitations of our study lay in the cross‐sectional design. A longitudinal design is preferable to track the progression of different PD subtypes. When it come to the effect of handedness on the dominant hemisphere, we only considered right‐handed subjects. The left‐handed subjects should be included in future studies. It should also be mentioned that MMSE scale was applied for reflecting cognition in PD patients, which only provides us a rough estimate of the cognition decline for there is still a ceiling effect. Therefore, it would be better to use other specific instruments such as the Montreal Cognitive Assessment (MoCA)^59^ or comprehensive cognitive domain assessments^60^ in future studies. But in this study, MMSE was insisted because it is a simple and universally applied measurement that is more suitable for less‐educated people and can be easily and rapidly acquired in the office.

## 5 CONCLUSIONS

In summary, our findings suggested that different combinations of laterality and motor types had diverse clinical characteristics; thereinto, LAR subtype had the worst motor and cognitive performance and the severer damage in the right cingulate region might be the underlying basis. This study underscores the importance of classifying PD subtypes based on both the side and type of motor symptom for clinical intervention and research to optimize behavioral outcomes in the future.

## AUTHOR CONTRIBUTIONS

J.C. and J.W. developed and designed the study concept; X.J. coordinated and collected the data; C.Z. and J.C. conducted the data analyses; J.C. wrote the initial draft, and it was revised by H.W., C.Z., and M.Z.; all authors confirmed the final version.

## CONFLICT OF INTEREST

The authors declare no competing interests.

## Data Availability

The data supporting the findings of this study are available from the corresponding author upon reasonable request. They are not publicly available due to ethical restrictions.

## References

[1] Armstrong MJ , Okun MS . Diagnosis and treatment of Parkinson disease: a review. JAMA. 2020;323(6):548‐560. 32044947 10.1001/jama.2019.22360

[2] Kalia LV , Lang AE . Parkinson's disease. Lancet. 2015; 386(9996):896‐912. 25904081 10.1016/S0140-6736(14)61393-3

[3] Jankovic J . Parkinson's disease: clinical features and diagnosis. J Neurol Neurosurg Psychiatry. 2008;79(4):368‐376. 18344392 10.1136/jnnp.2007.131045

[4] Jankovic J , Tan EK . Parkinson's disease: etiopathogenesis and treatment. J Neurol Neurosurg Psychiatry. 2020;91(8):795‐808. 32576618 10.1136/jnnp-2019-322338

[5] Marras C , Chaudhuri KR . Nonmotor features of Parkinson's disease subtypes. Mov Disord. 2016;31(8):1095‐1102. 26861861 10.1002/mds.26510

[6] Rajput AH , Rajput ML , Ferguson LW , Rajput A . Baseline motor findings and Parkinson disease prognostic subtypes. Neurology. 2017;89(2):138‐143. 28592451 10.1212/WNL.0000000000004078 PMC5501934

[7] Rajput AH , Voll A , Rajput ML , Robinson CA , Rajput A . Course in Parkinson disease subtypes: a 39‐year clinicopathologic study. Neurology. 2009;73(3):206‐212. 19620608 10.1212/WNL.0b013e3181ae7af1

[8] Thenganatt MA , Jankovic J . Parkinson disease subtypes. JAMA Neurol. 2014;71(4):499‐504. 24514863 10.1001/jamaneurol.2013.6233

[9] Fereshtehnejad SM , Postuma RB . Subtypes of Parkinson's disease: what do they tell us about disease progression? Curr Neurol Neurosci Rep. 2017;17(4):34. 28324303 10.1007/s11910-017-0738-x

[10] Djaldetti R , Ziv I , Melamed E . The mystery of motor asymmetry in Parkinson's disease. Lancet Neurol. 2006;5(9):796‐802. 16914408 10.1016/S1474-4422(06)70549-X

[11] Miller‐Patterson C , Buesa R , McLaughlin N , Jones R , Akbar U , Friedman JH . Motor asymmetry over time in Parkinson's disease. J Neurol Sci. 2018;393:14‐17. 30096567 10.1016/j.jns.2018.08.001

[12] Bohnen NI , Albm RL , Koeppe RA , et al. Positron emission tomography of monoaminergic vesicular binding in aging and Parkinson disease. J Cereb Blood Flow Metab. 2006;26(9):1198‐1212. 16421508 10.1038/sj.jcbfm.9600276

[13] Fiorenzato E , Antonini A , Bisiacchi P , Weis L , Biundo R . Asymmetric dopamine transporter loss affects cognitive and motor progression in Parkinson's disease. Mov Disord. 2021;36(10):2303‐2313. 34124799 10.1002/mds.28682 PMC8596815

[14] Cubo E , Martin PM , Martin‐Gonzalez JA , Rodriguez‐Blazquez C , Kulisevsky J , Members EG . Motor laterality asymmetry and nonmotor symptoms in Parkinson's disease. Mov Disord. 2010;25(1):70‐75. 20014110 10.1002/mds.22896

[15] Riederer P , Sian‐Hulsmann J . The significance of neuronal lateralisation in Parkinson's disease. J Neural Transm. 2012;119(8):953‐962. 22367437 10.1007/s00702-012-0775-1

[16] Katzen HL , Levin BE , Weiner W . Side and type of motor symptom influence cognition in Parkinson's disease. Mov Disord. 2006;21(11):1947‐1953. 16991155 10.1002/mds.21105

[17] Rodriguez‐Violante M , Cervantes‐Arriaga A , Villar‐Velarde A , Corona T . Relationship between the type and side of motor symptoms with the prevalence of non‐motor symptoms in Parkinson's disease. Neurologia. 2011;26(6):319‐324. 21315490 10.1016/j.nrl.2010.12.008

[18] Baumann CR , Held U , Valko PO , Wienecke M , Waldvogel D . Body side and predominant motor features at the onset of Parkinson's disease are linked to motor and nonmotor progression. Mov Disord. 2014;29(2):207‐213. 24105646 10.1002/mds.25650

[19] Seichepine DR , Neargarder S , Davidsdottir S , Reynolds GO , Cronin‐Golomb A . Side and type of initial motor symptom influences visuospatial functioning in Parkinson's disease. J Parkinsons Dis. 2015;5(1):75‐83. 25311203 10.3233/JPD-140365 PMC4593241

[20] Jankovic J , McDermott M , Carter J , et al. Variable expression of Parkinson's disease: a base‐line analysis of the DATATOP cohort. The Parkinson Study Group. Neurology. 1990;40(10):1529‐1534. 2215943 10.1212/wnl.40.10.1529

[21] Alves G , Larsen JP , Emre M , Wentzel‐Larsen T , Aarsland D . Changes in motor subtype and risk for incident dementia in Parkinson's disease. Mov Disord. 2006;21(8):1123‐1130. 16637023 10.1002/mds.20897

[22] Munhoz RP , Espay AJ , Morgante F , et al. Long‐duration Parkinson's disease: role of lateralization of motor features. Parkinsonism Relat Disord. 2013;19(1):77‐80. 22858180 10.1016/j.parkreldis.2012.07.008 PMC3635808

[23] Riederer P , Jellinger KA , Kolber P , Hipp G , Sian‐Hulsmann J , Kruger R . Lateralisation in Parkinson disease. Cell Tissue Res. 2018;373(1):297‐312. 29656343 10.1007/s00441-018-2832-z

[24] Tomer R , Levin BE , Weiner WJ . Side of onset of motor symptoms influences cognition in Parkinson's disease. Ann Neurol. 1993;34(4):579‐584. 8215246 10.1002/ana.410340412

[25] Lee EY , Sen S , Eslinger PJ , et al. Side of motor onset is associated with hemisphere‐specific memory decline and lateralized gray matter loss in Parkinson's disease. Parkinsonism Relat Disord. 2015;21(5):465‐470. 25749355 10.1016/j.parkreldis.2015.02.008 PMC4424064

[26] Mori S , Zhang J . Principles of diffusion tensor imaging and its applications to basic neuroscience research. Neuron. 2006;51(5):527‐539. 16950152 10.1016/j.neuron.2006.08.012

[27] Boonstra JT , Michielse S , Temel Y , Hoogland G , Jahanshahi A . Neuroimaging detectable differences between Parkinson's disease motor subtypes: a systematic review. Mov Disord Clin Pract. 2021;8(2):175‐192. 33553487 10.1002/mdc3.13107 PMC7853198

[28] Rosenberg‐Katz K , Herman T , Jacob Y , Giladi N , Hendler T , Hausdorff JM . Gray matter atrophy distinguishes between Parkinson disease motor subtypes. Neurology. 2013;80(16):1476‐1484. 23516323 10.1212/WNL.0b013e31828cfaa4 PMC3662357

[29] Barbagallo G , Caligiuri ME , Arabia G , et al. Structural connectivity differences in motor network between tremor‐dominant and nontremor Parkinson's disease. Hum Brain Mapp. 2017;38(9):4716‐4729. 28631404 10.1002/hbm.23697 PMC6866900

[30] Wen MC , Heng HSE , Lu Z , et al. Differential white matter regional alterations in motor subtypes of early drug‐Naive Parkinson's disease patients. Neurorehabil Neural Repair. 2018;32(2):129‐141. 29347868 10.1177/1545968317753075

[31] Kim JS , Yang JJ , Lee JM , Youn J , Kim JM , Cho JW . Topographic pattern of cortical thinning with consideration of motor laterality in Parkinson disease. Parkinsonism Relat Disord. 2014;20(11):1186‐1190. 25231669 10.1016/j.parkreldis.2014.08.021

[32] Hughes AJ , Daniel SE , Kilford L , Lees AJ . Accuracy of clinical diagnosis of idiopathic Parkinson's disease: a clinico‐pathological study of 100 cases. J Neurol Neurosurg Psychiatry. 1992;55(3):181‐184. 1564476 10.1136/jnnp.55.3.181 PMC1014720

[33] Huang P , Tan YY , Liu DQ , et al. Motor‐symptom laterality affects acquisition in Parkinson's disease: a cognitive and functional magnetic resonance imaging study. Mov Disord. 2017;32(7):1047‐1055. 28712121 10.1002/mds.27000

[34] Seghier ML . Laterality index in functional MRI: methodological issues. Magn Reson Imaging. 2008;26(5):594‐601. 18158224 10.1016/j.mri.2007.10.010 PMC2726301

[35] Ham JH , Lee JJ , Kim JS , Lee PH , Sohn YH . Is dominant‐side onset associated with a better motor compensation in Parkinson's disease? Mov Disord. 2015;30(14):1921‐1925. 26408124 10.1002/mds.26418

[36] Kang GA , Bronstein JM , Masterman DL , Redelings M , Crum JA , Ritz B . Clinical characteristics in early Parkinson's disease in a central California population‐based study. Mov Disord. 2005;20(9):1133‐1142. 15954133 10.1002/mds.20513 PMC3643967

[37] Guan X , Zeng Q , Guo T , et al. Disrupted functional connectivity of basal ganglia across tremor‐dominant and akinetic/rigid‐dominant Parkinson's disease. Front Aging Neurosci. 2017;9:360. 29163141 10.3389/fnagi.2017.00360 PMC5673841

[38] Fischl B , Dale AM . Measuring the thickness of the human cerebral cortex from magnetic resonance images. Proc Natl Acad Sci U S A. 2000;97(20):11050‐11055. 10984517 10.1073/pnas.200033797 PMC27146

[39] Fischl B , Salat DH , Busa E , et al. Whole brain segmentation. Neuron. 2002;33(3):341‐355. 11832223 10.1016/s0896-6273(02)00569-x

[40] Smith SM , Jenkinson M , Johansen‐Berg H , et al. Tract‐based spatial statistics: voxelwise analysis of multi‐subject diffusion data. NeuroImage. 2006;31(4):1487‐1505. 16624579 10.1016/j.neuroimage.2006.02.024

[41] Wakana S , Jiang H , Nagae‐Poetscher LM , van Zijl PC , Mori S . Fiber tract‐based atlas of human white matter anatomy. Radiology. 2004;230(1):77‐87. 14645885 10.1148/radiol.2301021640

[42] van Dyck CH , Seibyl JP , Malison RT , et al. Age‐related decline in dopamine transporters: analysis of striatal subregions, nonlinear effects, and hemispheric asymmetries. Am J Geriatr Psychiatry. 2002;10(1):36‐43. 11790633

[43] Kempster PA , Gibb WR , Stern GM , Lees AJ . Asymmetry of substantia nigra neuronal loss in Parkinson's disease and its relevance to the mechanism of levodopa related motor fluctuations. J Neurol Neurosurg Psychiatry. 1989;52(1):72‐76. 2709038 10.1136/jnnp.52.1.72 PMC1032660

[44] Haaxma CA , Helmich RC , Borm GF , Kappelle AC , Horstink MW , Bloem BR . Side of symptom onset affects motor dysfunction in Parkinson's disease. Neuroscience. 2010;170(4):1282‐1285. 20723583 10.1016/j.neuroscience.2010.07.030

[45] Rajput AH , Sitte HH , Rajput A , Fenton ME , Pifl C , Hornykiewicz O . Globus pallidus dopamine and Parkinson motor subtypes: clinical and brain biochemical correlation. Neurology. 2008;70(16 Pt 2):1403‐1410. 18172064 10.1212/01.wnl.0000285082.18969.3a

[46] Kaasinen V , Kinos M , Joutsa J , Seppanen M , Noponen T . Differences in striatal dopamine transporter density between tremor dominant and non‐tremor Parkinson's disease. Eur J Nucl Med Mol Imaging. 2014;41(10):1931‐1937. 24867256 10.1007/s00259-014-2796-5

[47] Selikhova M , Williams DR , Kempster PA , Holton JL , Revesz T , Lees AJ . A clinico‐pathological study of subtypes in Parkinson's disease. Brain. 2009;132(Pt 11):2947‐2957. 19759203 10.1093/brain/awp234

[48] Dewey RB Jr , Taneja A , McClintock SM , et al. Motor symptoms at onset of Parkinson disease and risk for cognitive impairment and depression. Cogn Behav Neurol. 2012;25(3):115‐120. 22960435 10.1097/WNN.0b013e31826dfd62 PMC3477612

[49] Paus T . Primate anterior cingulate cortex: where motor control, drive and cognition interface. Nat Rev Neurosci. 2001;2(6):417‐424. 11389475 10.1038/35077500

[50] Margulies DS , Kelly AM , Uddin LQ , Biswal BB , Castellanos FX , Milham MP . Mapping the functional connectivity of anterior cingulate cortex. NeuroImage. 2007;37(2):579‐588. 17604651 10.1016/j.neuroimage.2007.05.019

[51] Schaeffer DJ , Gilbert KM , Ghahremani M , Gati JS , Menon RS , Everling S . Intrinsic functional clustering of anterior cingulate cortex in the common marmoset. NeuroImage. 2019;186:301‐307. 30419289 10.1016/j.neuroimage.2018.11.005

[52] Heilbronner SR , Haber SN . Frontal cortical and subcortical projections provide a basis for segmenting the cingulum bundle: implications for neuroimaging and psychiatric disorders. J Neurosci. 2014;34(30):10041‐10054. 25057206 10.1523/JNEUROSCI.5459-13.2014 PMC4107396

[53] Bozkurt B , Yagmurlu K , Middlebrooks EH , et al. Microsurgical and tractographic anatomy of the supplementary motor area complex in humans. World Neurosurg. 2016;95:99‐107. 27476690 10.1016/j.wneu.2016.07.072

[54] Vogt BA . Cingulate cortex in Parkinson's disease. Handb Clin Neurol. 2019;166:253‐266. 31731914 10.1016/B978-0-444-64196-0.00013-3

[55] de Schipper LJ , van der Grond J , Marinus J , Henselmans JML , van Hilten JJ . Loss of integrity and atrophy in cingulate structural covariance networks in Parkinson's disease. Neuroimage Clin. 2017;15:587‐593. 28652971 10.1016/j.nicl.2017.05.012 PMC5477092

[56] Kamagata K , Motoi Y , Abe O , et al. White matter alteration of the cingulum in Parkinson disease with and without dementia: evaluation by diffusion tensor tract‐specific analysis. AJNR Am J Neuroradiol. 2012;33(5):890‐895. 22241380 10.3174/ajnr.A2860 PMC7968830

[57] Zheng Z , Shemmassian S , Wijekoon C , Kim W , Bookheimer SY , Pouratian N . DTI correlates of distinct cognitive impairments in Parkinson's disease. Hum Brain Mapp. 2014;35(4):1325‐1333. 23417856 10.1002/hbm.22256 PMC3664116

[58] Stenset V , Bjornerud A , Fjell AM , et al. Cingulum fiber diffusivity and CSF T‐tau in patients with subjective and mild cognitive impairment. Neurobiol Aging. 2011;32(4):581‐589. 19428143 10.1016/j.neurobiolaging.2009.04.014

[59] Skorvanek M , Goldman JG , Jahanshahi M , et al. Global scales for cognitive screening in Parkinson's disease: Critique and recommendations. Mov Disord. 2018;33(2):208‐218. 29168899 10.1002/mds.27233

[60] Dubois B , Burn D , Goetz C , et al. Diagnostic procedures for Parkinson's disease dementia: recommendations from the movement disorder society task force. Mov Disord. 2007;22(16):2314‐2324. 18098298 10.1002/mds.21844

